# Editorial: Reviews in hematologic malignancies: 2023

**DOI:** 10.3389/fonc.2024.1412843

**Published:** 2024-05-02

**Authors:** Billy Michael Chelliah Jebaraj, Michael Liew, Felix Seyfried

**Affiliations:** ^1^ Division of Chronic Lymphocytic Leukemia, Department of Internal Medicine III, Ulm University Medical Center, Ulm, Germany; ^2^ ARUP Institute for Clinical and Experimental Pathology^®^ , Salt Lake City, UT, United States; ^3^ Department of Pediatrics and Adolescent Medicine, Ulm University Medical Center, Ulm, Germany

**Keywords:** hematologic malignancies, reviews, molecular mechanisms, disease pathology, lymphoma, multiple myeloma, clinical trends

## Editorial

The research being applied to hematological malignancies is as diverse and heterogeneous as the disease itself, all moving towards the ultimate goal of better therapeutic outcomes. In this article Research Topic, molecular pathway targets for therapy and the tumor microenvironment are highlighted for further research. Along with current clinical trends, a focus on hematological composite tumors and treatment guidance for developing countries like India are provided. Lastly, this Research Topic would not be complete without discussing next generation sequencing (NGS) and machine learning, which revolutionize molecular characterization, diagnostics and treatment optimization.

### Molecular pathways

Exploring molecular pathways to prevent tumor progression is an important area of research. For instance, Schmid and Hobeika in their review on B-cell receptor (BCR) signaling in chronic lymphocytic leukemia (CLL) present a detailed account on the biology of various components of the signaling pathway. The importance of BCR signaling for CLL is exemplified by the clinical success of inhibitors targeting Bruton’s tyrosine kinase (BTK), a key component of the pathway. The authors also present an update on pre-clinical and clinical efficacy of next-generation inhibitors of BCR, mechanisms that mediate resistance to these inhibitors and strategies to overcome resistance. Uncontrolled cell proliferation is a hallmark of cancers and William et al. explore the role of SKP2 (S-Phase kinase-related protein 2), in promoting cell cycle progression through ubiquitin-mediated degradation of cell cycle regulator proteins. Overexpression of *SKP2* has been associated with poor prognosis in solid tumors. This also appears to be the case in hematological malignancies. In addition, *SKP2* overexpression is associated with drug resistance in hematological malignancies. Research is needed to develop novel inhibitors of SKP2 to overcome the drug resistance.

A small nucleocytoplasmic shuttling protein myeloid leukemia factor 1 (MLF1) is known to act as a ‘double-edged sword’ in a context dependent manner. Li et al. review the role of MLF1 in myeloid neoplasms. The precise role of MLF1 is poorly understood, but it has been implicated in the development of acute myeloid leukemia (AML) as well as myelodysplastic syndrome (MDS). For example, high expression of MLF1 is associated with poor prognosis in AML, but in a drosophila model of leukemia MLF1 reduced RUX1-ETO-dependent leukemia cell proliferation. More research into the role of MLF1 in the normal functioning of a cell will help determine if it is a candidate for cancer therapeutics. On the other hand, long non-coding RNAs (lncRNAs) are described as being involved in a plethora of processes including tumorigenesis. However, an in-depth understanding of each lncRNA is lacking. Nylund et al. comprehensively summarize recent studies supporting the clinical relevance of lncRNAs in multiple myeloma (MM). Sequencing analyses have provided evidence that lncRNAs contribute to disease development, treatment resistance, and patient prognosis in MM. lncRNAs have been shown to modulate chromatin remodeling and to impact gene expression. Targeting lncRNAs is emerging as a possible therapeutic approach for cancer, including MM.

Studying cell death mechanisms is important for refining cancer therapy. Ferroptosis is a form of cell death which is different from autophagy, apoptosis and necrosis and Chen et al. review its role in leukemia. Ferroptosis is characterized by iron-dependent lipid peroxidation and reactive oxygen species (ROS) accumulation, which eventually becomes fatal to a cell. If ferroptosis could be enhanced specifically in leukemic cells, this could serve as a possible therapeutic. Several studies have demonstrated that current therapies do induce ferroptosis, but some cancers are able to evade cell death. Research is needed to investigate the specific mechanisms that prevent ferroptosis.

### Tumor microenvironment

The tumor microenvironment plays an important role in the pathogenesis and progression of various hematological malignancies. In their review, Ding et al. summarize the recent findings on a crucial component of the tumor microenvironment called cancer-associated fibroblasts (CAFs). Various cells of mesenchymal stem cell origin can be reprogrammed into activated CAFs by tumors. The CAFs in-turn support tumor growth, drug resistance and metastasis. The authors highlight that a comprehensive understanding of CAFs in hematologic cancer would be important for innovative and next-generation cancer drug design. Extracellular vesicles (EVs) are capable of mediating complex crosstalk between tumor cells, as well as their microenvironment. EVs shuttle various proteins, lipids and nucleic acids between cells. Bernardi et al. in their review compiled key findings on the role of EVs in chronic myeloid leukemia (CML). Of importance, the authors emphasize on translational aspects such as the potential value of EVs for monitoring minimal residual disease (MRD), as biomarkers for optimizing treatments, and to analyze therapy efficacy. They also present from the literature, interesting prospects to reprogram EVs as targeted drug delivery vehicles for CML treatment.

### Clinical trends

In the clinical setting, use of tyrosine kinase inhibitors (TKIs) substantially changed the treatment perspective of CML, but chronic use is associated with adverse events. Cheng et al. discuss dose optimization strategies for TKIs in chronic myeloid leukemia (CML). The authors summarize recent clinical trials and real-life practices in which an increasing number of CML patients have undergone a dose optimization strategy involving dose reduction and discontinuation of TKI therapy. They discuss how treatment discontinuation has now emerged as a therapeutic goal for CML patients with a deep molecular response and has proven to be feasible in about half of patients.

Survival of patients with acute lymphoblastic leukemia (ALL) has greatly improved in the recent decade. However, for developing countries like India, it remains a challenge due to direct costs such as the financial cost of treatment and indirect costs such as the loss of productive years of the patient and caregiver and the rise of more resistant forms of the disease due to difficulties in timely treatment delivery. Mathews et al. describe how a panel of 15 actively practicing clinicians developed a consensus document for B-ALL management to offer assistance to Indian hematologists/oncologists. Strategies like this are very important to ensure that effective treatments are available to everyone.

The complexity of hematologic malignancies being managed clinically is illustrated by a case discussed by Gu et al. Composite lymphomas (CL) are an unusual type of hematologic malignancy, accounting for 1-4.7% of all lymphomas. Even more uncommon are CLs that comprise of both a B-cell and a T-cell tumor. The authors present their case of a mixture of diffuse large B cell lymphoma (DLBCL) and peripheral T-cell lymphoma, not otherwise specified (PTCL-NOS). The mechanism by which CL arises has not been elucidated, but hypotheses include virological and a specific mutation in a progenitor cell. These are a challenging class of lymphoma and will require further study to improve their poor prognosis. Hepatitis B virus (HBV) is the most common cause of liver disease worldwide and it is associated with lymphoma in endemic regions. Rosenberg et al. provide an overview of hepatitis B virus (HBV) infection in B-cell lymphoma. The authors emphasize the importance of systematic screening and preventive antiviral therapy for non-Hodgkin’s lymphoma (NHL) patients. The review summarizes studies showing a connection between HBV and lymphoma, particularly DLBCL. In addition, recent studies have revealed that HBV-positive DLBCL has distinct mutational signatures with differential outcomes.

In another setting of hematological disorders, Nassani et al. present an important clinical summary on the benefits and adverse effects of androgen therapy in different BMF syndromes. Androgens are an important class of molecules that are in clinical use for treating various bone marrow failure (BMF) syndromes since decades. Though their mechanism of action in stimulating hematopoiesis is unresolved, they continue to prove to be an important class of treatment for specific clinical scenarios. The authors give practical recommendations for use of androgens for BMF patients.

### NGS and deep learning

Over the past decade, substantial advances have been made in NGS technologies. Tomacinschii et al. provide a comprehensive review of recent developments in NGS for the diagnosis and clinical management of NHL patients. The data generated by NGS allows the identification of genetic markers specific to different subtypes, leading to a more accurate diagnosis and classification. The integration of genomic and transcriptomic data can improve the understanding of the mechanisms of tumor development and can help select the optimal therapy. DL and artificial intelligence (AI) are revolutionizing every field. Elsayed et al. review recent studies that examine the use of DL in the diagnosis of ALL, focusing on the analysis of bone marrow images. DL approaches, especially those using Convolutional Neural Networks (CNN) techniques, have achieved excellent results in classifying cancer cells. The authors propose that DL methods have high potential for reliable classification of ALL in a clinical context. Further models aim to combine both image analysis and genomic data, which could lead to improvements in ALL classification.

In conclusion, there are still avenues open for exploration to improve the treatment of hematological malignancies. As current therapies, clinical guidelines, and the use of AI continue to get refined and improved, they contribute to steadily improving outcomes for patients with hematological malignancies.

## Author contributions

BMCJ: Writing – original draft, Writing – review & editing. ML: Writing – original draft, Writing – review & editing. FS: Writing – original draft, Writing – review & editing.

